# Oxidative Stress Can Be Attenuated by 4-PBA Caused by High-Fat or Ammonia Nitrogen in Cultured Spotted Seabass: The Mechanism Is Related to Endoplasmic Reticulum Stress

**DOI:** 10.3390/antiox11071276

**Published:** 2022-06-28

**Authors:** Yanzou Dong, Lei Li, Tian Xia, Lina Wang, Liping Xiao, Nengshui Ding, Youlin Wu, Kangle Lu

**Affiliations:** 1 Key Laboratory of Healthy Mariculture for the East China Sea, Ministry of Agriculture and Rural Affairs, Fisheries College, Jimei University, Xiamen 361021, China; 201911908030@jmu.edu.cn (Y.D.); lileixlt@163.com (L.L.); 202011908013@jmu.edu.cn (T.X.); 2 Key Laboratory of Swine Nutrition and Feed Science of Fujian Province, Fujian Aonong Biological Science and Technology Group Co., Ltd., Zhangzhou 363000, China; wanglina@aonong.com.cn (L.W.); xiaoliping@aonong.com.cn (L.X.); dingnengshui@aonong.com.cn (N.D.)

**Keywords:** 4-phenylbutyric acid, endoplasmic reticulum stress, oxidative stress, high-fat diet, ammonia nitrogen exposure

## Abstract

Oxidative stress is a common phenomenon in aquaculture, which can be induced by nutritional or environmental factors. Generally, oxidative stress causes poor growth performance, metabolic dysregulation, and even the death of aquatic animals. To identify a nutritional intervention strategy, high-fat diet (HFD) feeding (Experiment I) and acute ammonia nitrogen challenge (Experiment II) tests were carried out. In Experiment I, HFD feeding significantly decreased the growth performance concomitantly with excessive fat deposition in the liver and abdomen. The addition of 4-PBA in the diet improved the excessive fat accumulation. The activities of antioxidative enzymes were suppressed, and the levels of lipid and protein peroxidation were increased, indicating that HFD feeding induced oxidative stress. The endoplasmic reticulum stress (ERs) related genes were downregulated in the HFD group. Under a transmission electron microscope (TEM), more swollen and dilated ER lumen could be observed. These results indicated that the HFD induced ERs activation. Although 4-PBA acted as a potent ERs inhibitor, as evidenced by the alleviated alterations of ERs molecules and the ER ultrastructure, the oxidative stress was also attenuated by 4-PBA. In Experiment II, dietary 4-PBA improved the tolerance to the acute ammonia nitrogen challenge, as lower mortality and serum aminotransferase activity was found. Further results showed that 4-PBA decreased the peroxidation content and attenuated ERs, thus confirming the correlation between oxidative stress and ERs. Our findings showed that dietary 4-PBA supplementation can attenuate oxidative stress induced by a HFD or acute ammonia challenge; the mechanism is related to its potent inhibition effect for ERs.

## 1. Introduction

Today, aquatic products are of significant nutritional interest for billions of people worldwide [1]. Furthermore, aquatic foods are regarded as ideal sources of quality protein and essential fatty acids, which could improve a range of human pathologies [2]. Over the past few decades, Chinese aquaculture production has been steadily pursuing growth, and it has become the fastest growing food production sector [3]; however, during the farming process, oxidative stress damage is ubiquitous, and it often seriously affects the growth performance, stress tolerance, and pathogen sensitivity of aquatic animals [4,5,6].

Oxidative stress occurs due to the disruptions between the generation of reactive oxygen species (ROS) and antioxidant defenses in living organisms [7]. Oxidative stress could cause damage to cells and tissues, and it mainly manifests as the peroxidation of biomacromolecules and cell apoptosis [8,9]. In general, many exogenous factors can induce oxidative stress, including oxidized fat and high-fat diets, ammonia nitrogen, and so on [10,11,12,13]. Fat plays a dominant role in energy storage and supply for animals, due to its high energy density [14,15]; hence, the high-fat diet is extensively used in fish cultures for its protein-sparing effect [14,16,17]. Ammonia is the main nitrogen-based catabolic product that is released in the aquaculture system. Recently, a high-density pattern emerged in the aquaculture industry, which has become the main trigger for the overproduction of ammonia nitrogen [18]. The high-fat diet feeding, and the ammonia nitrogen compounds, can induce oxidative stress by overproducing ROS, nitric oxide, and reactive nitrogen species in fish [19]; therefore, there is a pressing need to reveal the underlying physiological mechanisms of oxidative stress induced by high-fat diet feeding and ammonia nitrogen, and to develop antioxidant strategies.

Spotted seabass (*Lateolabrax maculatus*) is a carnivorous species with a high growth speed, flesh quality, and economic value [20]. It has become the second major cultured marine fish in China, with 195,246 tons being produced in 2020 [21]. The high-fat diet is widely used in its artificial rearing, which often leads to fat deposition and oxidative stress [10]. Moreover, the stocking density of spotted seabass is often high, with a production of >100 kg/m^3^; thus, it is a good model to study the oxidative stress caused by high-fat or ammonia nitrogen. Furthermore, the stress of the endoplasmic reticulum (ERs) is often found in fish that are exposed to high-fat diets or ammonia nitrogen [13,16,18,22]. The ER is also an important source of ROS, which accounts for approximately 25% of all ROS produced [23]. Recent findings showed that ERs-induced alterations in ROS production and scavenging mechanisms contribute to the worsening of oxidative stress [24]. Based on the above, the present study is conducted to evaluate the role of ERs in the process of oxidative stress and the regulation of 4-PBA on the oxidative stress of cultured fish.

## 2. Materials and Methods

### 2.1. Animals

Juvenile spotted seabass were purchased from a fish hatchery (Zhangzhou, China). Juveniles were first transported to the aquaculture system in Jimei University, and were kept in a 1500-L tank so that they could adapt to the experimental conditions for two weeks. In this period, fish were fed a Jia-kang brand commercial diet (Xiamen Jia-kang Foods Co., Ltd. Xiamen, China; 45% protein, 11% lipid) twice daily (8:00 and 17:00). The experimental conditions were maintained at the optimal water temperature (25–27 °C), pH (7.0–7.2), and level of dissolved oxygen (>6 mg/L).

### 2.2. Experimental Design

#### 2.2.1. Experiment I: The High-Fat Diet Feeding Study

Two experimental diets were produced with an 11% or 17% lipid level, which is regarded as normal fat diet (NFD) and high-fat diet (HFD), respectively. Moreover, 4-phenylbutyric acid (4-PBA) was added to the diet by adding 4-PBA to the HFD at a dose of 50 mg/kg (regarded 4-PBA). The formulation and proximate composition of experimental diets are shown in Table S1. The protocols of diet production and proximate composition determination were introduced in a previous study [21].

A total of 270 healthy fish of similar sizes (13.05 ± 0.15 g) were randomly distributed into nine 200-L tanks (30 fish per tank) in a recirculating aquaculture system (RAS). Freshwater was provided with a mechanical filtration system, UV treatment, and constant aeration. Fish were fed with a NFD, HFD, and 4-BPA, and each experimental diet was hand-fed to fish from three tanks to achieve visual satiety twice daily (8:00 and 17:00). After eight weeks of feeding, the fish were euthanized with 100 mg/L MS-222 (Sigma, St. Louis, MO, USA). Then, bodyweights were measured, and liver sampling was conducted for analysis, in accordance with the method described in our recent study [10]. The experimental conditions were consistent with the conditions maintained during the acclimation period.

#### 2.2.2. Experiment II: The Acute Ammonia Nitrogen Challenge Study

Fish from the same batch that were of similar initial sizes to the fish in Experiment I were distributed into six separate tanks (30 fish per tank). Three tanks of fish were fed a NFD and were regarded as control group, the other three were fed a NFD with 50 mg/kg 4-PBA supplementation, named the 4-PBA group. Feeding management is same as with Experiment I. After two weeks of feeding, an acute ammonia nitrogen challenge test was carried out. A stock solution of a high NH_4_Cl concentration (10 g/L) was prepared and subsequently added to each tank, the ammonia nitrogen concentration was adjusted to 95 mg/L, and the 48 h LC_50_ was carried out in our preliminary test. After 48 h, serum and liver samples were conducted. During this period, the ammonia concentrations of each tank were detected by nesslerization [25] every 6 h, and adjusted with a NH_4_Cl stock solution to maintain the desired concentration. One-third of the test water was replaced with fresh water every 12 h. Fish were fasting during this period.

### 2.3. Biochemical Assays

The contents of triglyceride (TG), protein carbonylation (PC), and malonaldehyde (MDA), and the activities of superoxide dismutase (SOD), catalase (CAT), and glutathione peroxidase (GSH-PX) were deleted in liver homogenate by using commercial kits (Nanjing JianCheng Bioengineering Institute, Nanjing, China). The activities of aspartate aminotransferase (AST) and alanine aminotransferase (ALT) were determined in serum in accordance with our recent study [26].

### 2.4. Liver Histology

Oil red O staining was used to evaluate fat deposition in the liver. Briefly, liver samples were fixed in a 10% formaldehyde solution for 24 h, then dehydrated in a 15% and 30% sugar solution at 4 °C. The dehydrated samples were embedded into an optimal cutting temperature (OCT) compound (Servicebio, Wuhan, China), and cut into 8 μm-thick sections using a freezing microtome (CRYOSTAR NX50, Thermo Scientific, Waltham, MA, USA). After that, sections were stained using oil red O and hematoxylin, then, they were observed and photographed under a microscope (DM5500B, Leica, Germany).

Transmission electron microscopy (TEM) analysis was conducted to observe hepatocellular ultrastructure. Samples were fixed in 2.5% glutaraldehyde solution overnight and post-fixed in osmic acid for 2 h at 4 °C. Then, the samples were dehydrated in gradient acetone solutions and embedded in epoxy resin. Ultrathin slices with a 60-nm thickness were produced, stained with uranyl acetate and lead citrate solutions, and observed under a TEM (Hitachi H-7650, Tokyo, Japan).

### 2.5. Gene Expression

A commercial kit (RC101-01, Vazyme Biotech Co., Ltd., Nanjing, China) was used to isolate the total RNA in the liver, according to the protocol provided by the manufacturer. Then, 1% agarose gel electrophoresis was carried out to investigate the RNA’s integrity. The agarose was obtained from LABLEAD. Inc. (Beijing, China). A NanoDrop™ One spectrophotometer (Thermo Scientific, Waltham, MA, USA) was used to determine the purity at 260/280 nm. First-strand cDNA was obtained from 1 μg of the total RNA, and a commercial kit was used to achieve this (R211-01, Vazyme Biotech Co., Ltd., Nanjing, China). The residual genomic DNA was erased using a gDNA wiper.

Real-time quantitative PCR (qPCR) was conducted in accordance with the method described in our recent study [27]. The primer sequences used in the present study were shown in Table S2. The relative expression levels of target genes were normalized by *β-actin* and calculated using the 2^−ΔΔCt^ method.

### 2.6. Statistical Analysis

Statistical analysis was performed using SPSS Statistics 20. For Experiment I, one-way ANOVA and Tuckey’s multiple range test were carried out to assess the differences between three treatments. Student’s *t*-test was utilized to evaluate the differences between two groups in Experiment II. Significance was set at *p* < 0.05 in both Experiment I and Experiment II, and all data were expressed as the means ± standard error (SE).

## 3. Results

### 3.1. Experiment I: The High-Fat Diet Feeding Study

#### 3.1.1. Growth and Fat Accumulation

**Figure 1:**
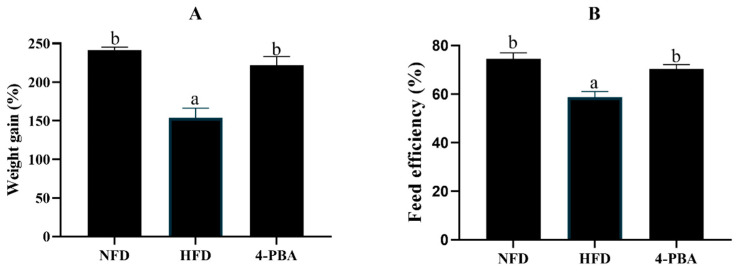
Weight gain (**A**) and feed efficiency (**B**) of spotted seabass (*L. maculatus*) that were fed the test diets for eight weeks. All values are exhibited as mean ± SE. The values with different superscripts (a, b) are significantly different at *p* < 0.05 (Tukey’s test). Weight gain = final body weight/initial body weight; Feed efficiency = wet weight gain/dry feed fed.

Fish that were fed the HFD showed a significantly lower weight gain (WG) and feed efficiency (FE) compared with fish that were fed the NFD. In addition, 4-PBA supplementation significantly improved the WG and FE (Figure 1).

**Figure 2:**
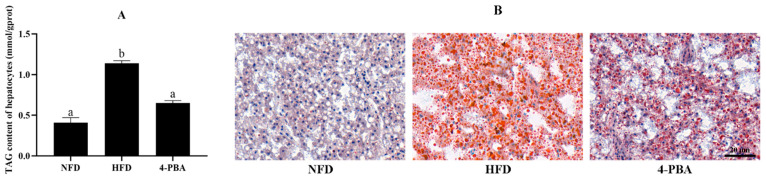
Hepatic TAG content (**A**) and oil red O-stained sections ((**B**) scale bar = 20 μm) of spotted seabass (*L. maculatus*) fed the test diets for eight weeks. All values are exhibited as mean ± SE. The values with different superscripts (a, b) are significantly different at *p* < 0.05 (Tukey’s test).

Moreover, fish that were fed the HFD exhibited excessive fat deposition compared with other groups. Severe liver steatosis was observed under oil red O straining. Hepatic triacylglycerol (TAG) content was also enhanced by the HFD. Furthermore, 4-PBA supplementation significantly reduced the oil red O-stained area and TAG content of hepatocytes (Figure 2).

#### 3.1.2. Oxidative Status

**Figure 3:**
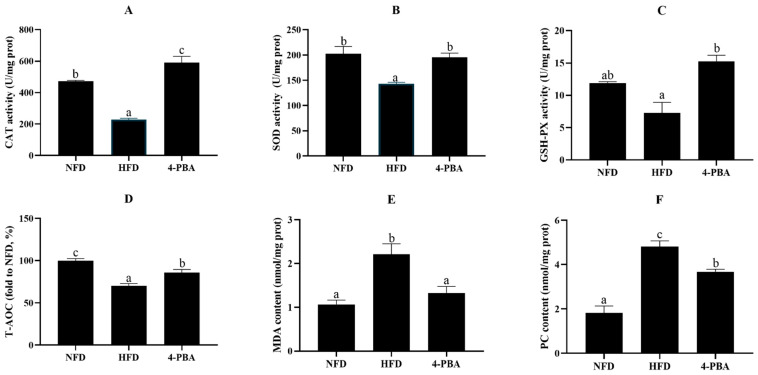
The activities of catalase (CAT: (**A**)), superoxide dismutase (SOD: (**B**)), glutathione peroxidase (GSH-PX: (**C**)), the level of total antioxidant capacity (T-AOC: (**D**)), and the content of malondialdehyde (MDA: (**E**)) and protein carbonylation (PC: (**F**)) in the livers of spotted seabass (*L. maculatus*) that were fed the test diets for eight weeks. All values are exhibited as mean ± SE. The values with different superscripts (a, b, c) are significantly different at *p* < 0.05 (Tukey’s test).

In the liver, the activities/level of CAT, SOD, GPX, and T-AOC of fish that were fed the HFD significantly decreased, whereas MDA and PC content significantly increased. The supplementation of 4-PBA dramatically elevated the activity/level of SOD, GPX, and T-AOC, and reduced the MDA and PC content (Figure 3).

#### 3.1.3. Endoplasmic Reticulum Stress

**Figure 4:**
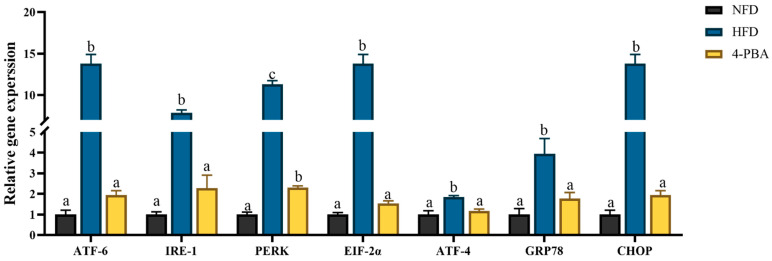
Relative expression levels of ERs-related genes in the livers of *L. maculatus* that were fed the test diets for eight weeks. All values are exhibited as mean ± SE. The values with different superscripts (a, b, c) are significantly different at *p* < 0.05 (Tukey’s test).

The expressions of ERS-related genes (*ATF-6*, *IRE-1*, *PERK*, *EIF-2α*, *ATF-4*, *GRP78*, and *CHOP*) were significantly upregulated by HFD feeding, and the application of 4-PBA significantly downregulated the expressions of these genes. (Figure 4).

**Figure 5:**
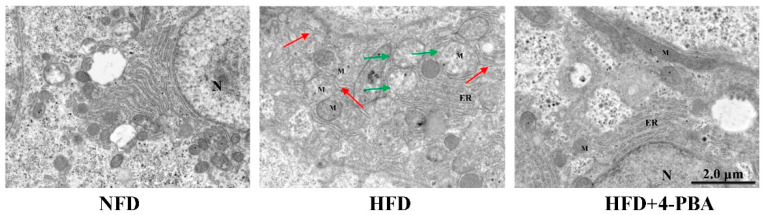
Hepatic transmission electron microscopy (TEM) images of *L. maculatus* that were fed the test diets for eight weeks. (N—nucleus; M—mitochondrion; green arrows—damaged mitochondria; red arrows—damaged endoplasmic reticulum).

Under the electron microscope, abnormalities were found in the livers of fish that were fed the HFD diet. In fish that were fed the NFD diet, the hepatocytes had stacks of rough endoplasmic reticulum (rER) that were concentrated around the nucleus and cell membrane borders; however, there was also a widespread swelling of the ER in fish that were fed the HFD diet. More mitochondria showed matrix losses in the HFD group, whereas this phenomenon was rare in the NFD and HFD+4-PBA groups (Figure 5).

### 3.2. Experiment II: The Ammonia Nitrogen Exposure Study

#### 3.2.1. The Survival and Liver Damage of Fish

**Figure 6:**
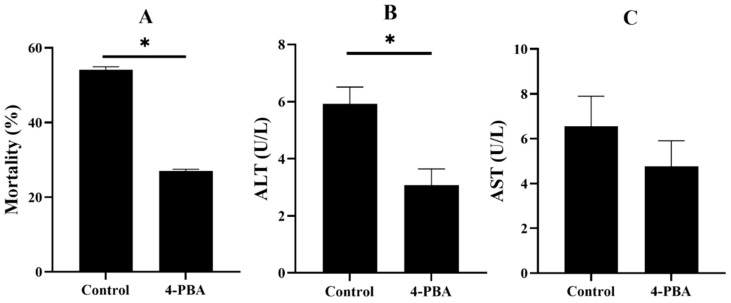
Mortality (**A**) and serum alanine aminotransferase (**B**) and aspartate aminotransferase (**C**) activities of *L. maculatus* after two weeks of being fed the test diets and 48 h of ammonia nitrogen exposure. All values are exhibited as mean ± SE. The values with different superscript (*) are significantly different at *p* < 0.05 (Student’s *t*-test).

After 48 h of ammonia nitrogen exposure, the mortality of the control group was about 50%, whereas the mortality of the 4-PBA group was significantly lower. The activity of ALT in the control group is significantly higher than that of the 4-PBA group, and the activity of AST exhibited the same trend but it did not cause a significant difference (Figure 6).

#### 3.2.2. Oxidative Status

**Figure 7:**
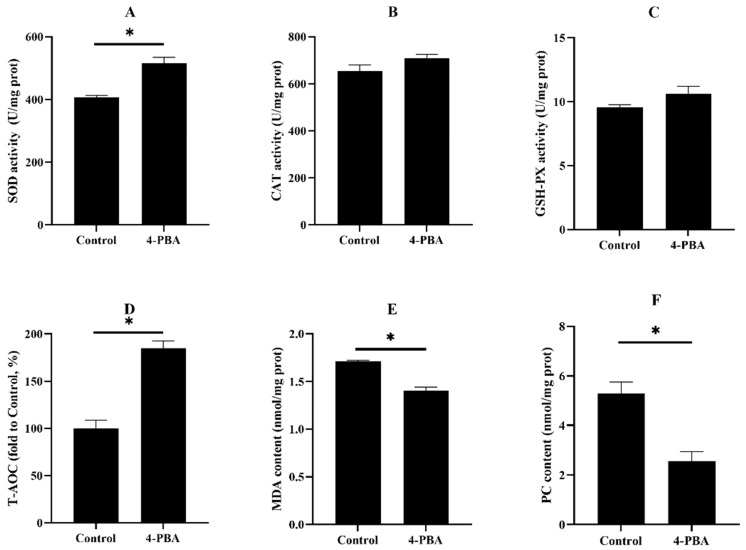
The activities of superoxide dismutase (SOD: (**A**)), catalase (CAT: (**B**)), glutathione peroxidase (GSH-PX (**C**)), the level of total antioxidant capacity (T-AOC: (**D**)), and the content of malondialdehyde (MDA: (**E**)) and protein carbonylation (PC: (**F**)) in the livers of *L. maculatus* after two weeks of being fed the test diets and 48 h of ammonia nitrogen exposure. All values are exhibited as mean ± SE. The values with different superscript (*) are significantly different at *p* < 0.05 (Student’s *t*-test).

The activities/levels of SOD and T-AOC in the fish that were fed the control diet significantly decreased, compared with the 4-PBA group. Although, the content of MDA and PC were much higher in the control group than in the 4-PBA group (Figure 7).

#### 3.2.3. Endoplasmic Reticulum Stress

**Figure 8:**
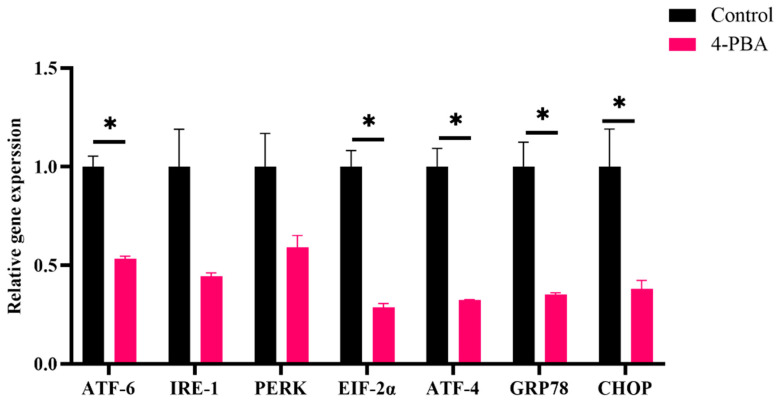
Relative expression levels of ERs-related genes in the livers of *L. maculatus* after two weeks of being fed the test diets and 48 h of ammonia nitrogen exposure. All values are exhibited as mean ± SE. The values with different superscript (*) are significantly different at *p* < 0.05 (Student’s *t*-test).

**Figure 9:**
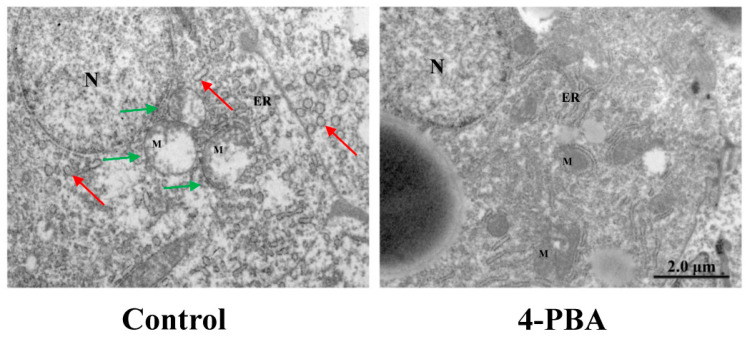
Hepatic transmission electron microscopy (TEM) images of *L. maculatus* after two weeks of being fed the test diets and 48 h of ammonia nitrogen exposure. (N—nucleus; M—mitochondrion; green arrows—damaged mitochondria; red arrows—damaged endoplasmic reticulum).

The expression levels of ERS-related genes (*CHOP*, *GRP78*, *ATF4*, *ATF6*, and *EIF-2α*) in the control group were remarkably higher than those in the 4-PBA group (Figure 8). The ultrastructural damage of the ER could be observed in both groups; however, in the control group, more ER lumens presented severe dilatation, and the ER network was fragmented (Figure 9).

## 4. Discussion

Oxidative stress refers to the excess reactive oxygen species (ROS) production that can be stimulated by environmental and nutritional factors [28]. ROS can induce the oxidative damage of biomacromolecules and cellular membrane systems [29]. Emerging evidence also indicates that ROS often initiate the inflammation response and apoptosis [30,31].

High fat intake could cause excess fat accumulation and induce oxidative stress in fish [27,32]. Moreover, the the activities of antioxidative enzymes (CAT, SOD, and GSH-PX) decreased, and then, peroxidation occurred. In the present study, the increased MDA and PC levels indicated that HFD feeding induced oxidative stress.

The endoplasmic reticulum (ER) is composed of a complex membrane system and is susceptible to attacking ROS [33]. As the main site of protein synthesis, folding, modification, and secretion, oxidative damage to the ER often induces protein unfolding or/and misfolding [34]. The aggregation of unfolded/misfolded proteins activates endoplasmic reticulum stress (ERs) [35]. Previous studies have demonstrated that ERs activation reiles on unfolded protein response (UPR) pathways [36]. As it is downstream of UPR, CHOP plays a vital role in ERs-mediated cytotoxicity, given its contributor role to apoptosis [37]. These molecules of the UPR pathway are well accepted as biomarkers of ERs. In this study, the expressions of ERs-related genes (*ATF-6*, *IRE-1*, *PERK*, *Eif-2α*, *ATF-4*, *GRP78*, and *CHOP*) were upregulated through HFD feeding. Moreover, swollen endoplasmic reticula were observed under TEM. These results indicated that HFD feeding activated ERs. Similar results have also been reported in other fish [16,22,38] and rodent models that were fed high-fat diets [39,40]. Furthermore, 4-Phenylbutyric acid (4-PBA) has an effect on misfolded and unfolded proteins; therefore, it can be used as a specific inhibitor of ERs [41,42]. The present results showed that dietary 4-PBA significantly decreased ERs induced by a HFD.

There are many proteins involved in the synthesis and export of lipids that are folded or/and bounded at the ER. Hence, the ER also plays a key role in the homeostasis of lipid metabolism [43]. The overload of lipid metabolism leads to continuous ERs, which leads to lipid synthesis as a result of the protein metabolism in the ER [43,44]. In the present study, 4-PBA exhibited fat lowering effects that are dependent on ER metabolism remolding. The fat-lowering effect of 4-PBA also contributed to the alleviation of oxidative stress [45].

It is reported that there is an interplay between ERs and oxidative stress [23]. The overexpression of UPR components, such as CHOP and ATF4, directly contribute to ROS synthesis in the ER [33]. Moreover, the impaired ER can translate Ca^2+^ to mitochondria through the calcium release channel and mitochondrial-associated membranes, which exaggerates the production of ROS via the electron transport chain (ETC) [46]. In the present study, the expression of *CHOP* and *ATF4* were downregulated by dietary 4-PBA. Moreover, the analysis of TEM showed that HFD-induced mitochondrial damage was also alleviated. Based on these phenomena, we postulate that 4-PBA also inhibits ROS formation in both the ER and mitochondria.

There are many factors in the aquaculture environment that can be triggers of oxidative stress [6,47]. Notably, the highly intensive aquaculture industry has boomed in recent decades, as there has been a high demand for fish products [48]. Thus, fish often suffer ammonia nitrogen stress in the highly intensive culture [12]. The high ammonia nitrogen concentration can cause serious tissue damage and high mortality, which is a high-risk factor during fish farming [49,50,51]. Ammonia nitrogen stress can rapidly gather in the blood and tissue of fish and act as a potent cause of oxidative stress [52]. Fish that were fed the 4-PBA supplementation diet had a lower mortality rate and serum transaminases activities after the acute ammonia nitrogen challenge. This indicates that dietary 4-PBA can improve the tolerance of fish to ammonia stress. Further analysis showed that 4-PBA can enhance antioxidative abilities and reduce the peroxidation of proteins and lipids. Moreover, 4-PBA downregulated the gene expression of UPR factors. This indicates that 4-PBA supplementation exhibits the protective effect of ER homeostasis in fish that are exposed to high ammonia nitrogen concentrations.

## 5. Conclusions

In summary, the present study showed that ERs played an important role in the oxidative stress induced by a HFD or stress caused by high ammonia nitrogen concentrations. Furthermore, 4-PBA can attenuate the stress of the ER and excess fat deposition caused by the HFD feeding. Moreover, the supplementation of 4-PBA can increase the tolerance of fish that have suffered ammonia stress.

## Data Availability

The data presented in this study are available on request from the corresponding author.

## Supplementary Materials

The following supporting information can be downloaded at: https://www.mdpi.com/article/10.3390/antiox11071276/s1, Table S1: Formulation and Proximate Composition of Experimental Diets; Table S2: Sequences of Primers Used for RT-qPCR.
