# Supplementary material for: Oxidative Stress Can Be Attenuated by 4-PBA Caused by High-Fat or Ammonia Nitrogen in Cultured Spotted Seabass: The Mechanism Is Related to Endoplasmic Reticulum Stress
Source: Antioxidants (Basel). 2022 Jun 28;11(7):1276. doi: 10.3390/antiox11071276 (PMC9312264; doi:10.3390/antiox11071276)
Supplement: Supplementary file 1 [file antioxidants-11-01276-s001.zip › antioxidants-1772451-supplementary.pdf]

Table S1. Formulation and proximate composition of experimental diets.

Ingredients (g/kg)	LFD	HFD
Fish meal	268.5	268.5
Poultry meal	84.7	84.7
Wheat gluten	73	87.5
Soybean meal	250	250
α-starch	30	30
Wheat flour	201.1	125.1
Fish oil	19.3	50
Soybean oil	19.3	50
Soybean Lecithin	20	20
Premix1	13.5	13.5
Calcium biphosphate	20.8	20.8
Proximate composition (g/kg)		
Moisture	49.3	37.4
Protein	438.6	436.9
Lipid	110.7	168.6
Ash	85.7	84.8

[1]Premix supplied the following minerals (g/kg) and vitamins (IU or mg/kg): CuSO4·5H2O, 2.0 g; $FeSO_4 \cdot 7H_2O$, 25 g; $ZnSO_4 \cdot 7H_2O$, 22 g; $MnSO_4$·4H2O, 7 g; Na_2SeO_3, 0.04 g; KI, 0.026 g; $CoCl_2 \cdot 6H_2O$, 0.1 g; Vitamin A, 900000 IU; Vitamin D, 200000 IU; Vitamin E, 4500 mg; Vitamin K3, 220 mg; Vitamin B1, 320 mg; Vitamin B2, 1090 mg; Niacin, 2800 mg; Vitamin B5, 2000 mg; Vitamin B6, 500 mg; Vitamin B12, 1.6 mg; Vitamin C, 5000 mg; Pantothenate, 1000 mg; Folic acid, 165 mg; Choline, 60000 mg.

Table S2. Sequences of primers used for RT-qPCR.

Target gene	Primer sequence (5'-3')	Amplification efficiency
β-actin[1]	F: TCGAGCACGGTATTGTGACC<br>R: TCAGGTGCAACTCTCAGCTC	99.87%
*GRP*78	F: GGGAGAAGAGGAGAAAGGTCTG<br>R: GCTTATCACCGCTCCGCTT	99.65%
CHOP	F: TGGGAGAAGAGGAGAAAGGTCT<br>R: CTTATCACCGCTCCGCTTGG	99.98%
PERK	F: GTTTTCACCCCAGCAAGCAG<br>R: AACCTTAGTGTCGGCCTTGG	99.94%
*IRE*1	F: AAAGGTTGTTCAGGGTGGCAT<br>R: GCAGCAATCAATCAACAAGCAAA	99.79%
ATF-6	F: AACGAGCACTTGAGGAGAGC<br>R: CAGACGCTCACTCCTTGTGT	94.60%
ATF-4	F: GTGTTGGTGCTTTGGGAACG<br>R: CATCAGACTCGCCCCTGTTT	99.79%
EIF-2*α*	F: CTCTCCACACGAGGTGCTTC<br>R: TCGTGGTCTTCTGGGTGGTA	99.66%
FAS	F: AAACTGAAGCCCTGTGTGCC<br>R: CACCCTGCCTATTACATTGCTC	98.80%
SREBP-1*c*	F: CCTCACTCTGCAGCCAATCA<br>R: CGTAGTCCCACCCTCAAACC	99.48%
ACC	F: AAGGCGGTGGTGATGGATTT<br>R: GGCCATGTCGCCTTTGTTTT	99.79%

grp78: glucose regulated protein 78; *chop*: C/EBP homology protein; *perk*: PKR-like endoplasmic reticulum kinase; *ire*1: inositol-requiring enzyme 1; *atf*-6: activating transcription factor 6; *atf*-4: activating transcription factor 4; *eif-2α*: eukaryotic initiation factor 2; *fas*: fatty acid synthetase; *serbp-1c*: sterol-regulation element binding protein-1c; *acc*: acetyl-coa carboxylase.

[1]Reference gene
